# *TMPRSS2:ERG*–directed radiosensitization: exploiting DNA repair rewiring in gene fusion–positive prostate cancer

**DOI:** 10.1172/JCI203657

**Published:** 2026-03-16

**Authors:** Xiaoju Wang, Arul M. Chinnaiyan

**Affiliations:** 1 Michigan Center for Translational Pathology,; 2 Department of Pathology,; 3 Rogel Cancer Center,; 4 Howard Hughes Medical Institute, and; 5 Department of Urology, University of Michigan, Ann Arbor, Michigan, USA.

## Abstract

The *TMPRSS2:ERG* gene fusion is a truncal oncogenic event in a large subset of prostate cancers, yet its clinical relevance has remained unclear. In this issue of the *JCI*, Köcher et al. have demonstrated that ERG overexpression in human prostate cancer cells rewired DNA double-strand break repair toward a poly(ADP-ribose) polymerase 1–dependent (PARP1-dependent) alternative end-joining pathway without disrupting canonical repair. This repair bias created a conditional dependency on PARP1 that was exposed by radiotherapy, rendering ERG-positive tumors selectively sensitive to PARP inhibition–mediated radiosensitization. The tumor-selective cytotoxic effect of combined PARP1 inhibition and irradiation was corroborated in human-derived prostate cancer organoids. These findings establish ERG as a predictive biomarker for precision radiotherapy and highlight a tumor-selective strategy to enhance radiotherapeutic efficacy in prostate cancer.

## Introduction

Prostate cancer is the most commonly diagnosed malignancy in men worldwide and a leading cause of cancer-related mortality (1). While localized disease is often curable, outcomes for patients with high-risk localized or locally advanced prostate cancer remain suboptimal despite multimodal therapy (2). Radiotherapy (RT), frequently combined with androgen deprivation therapy (ADT), remains a cornerstone of treatment across disease stages (3). However, the therapeutic index of RT is limited by its toxicity in normal tissue, motivating the development of tumor-selective radiosensitization strategies (4). In this context, tumor-intrinsic genomic alterations that reprogram DNA damage responses offer a compelling framework for precision RT.

**Figure 1:**
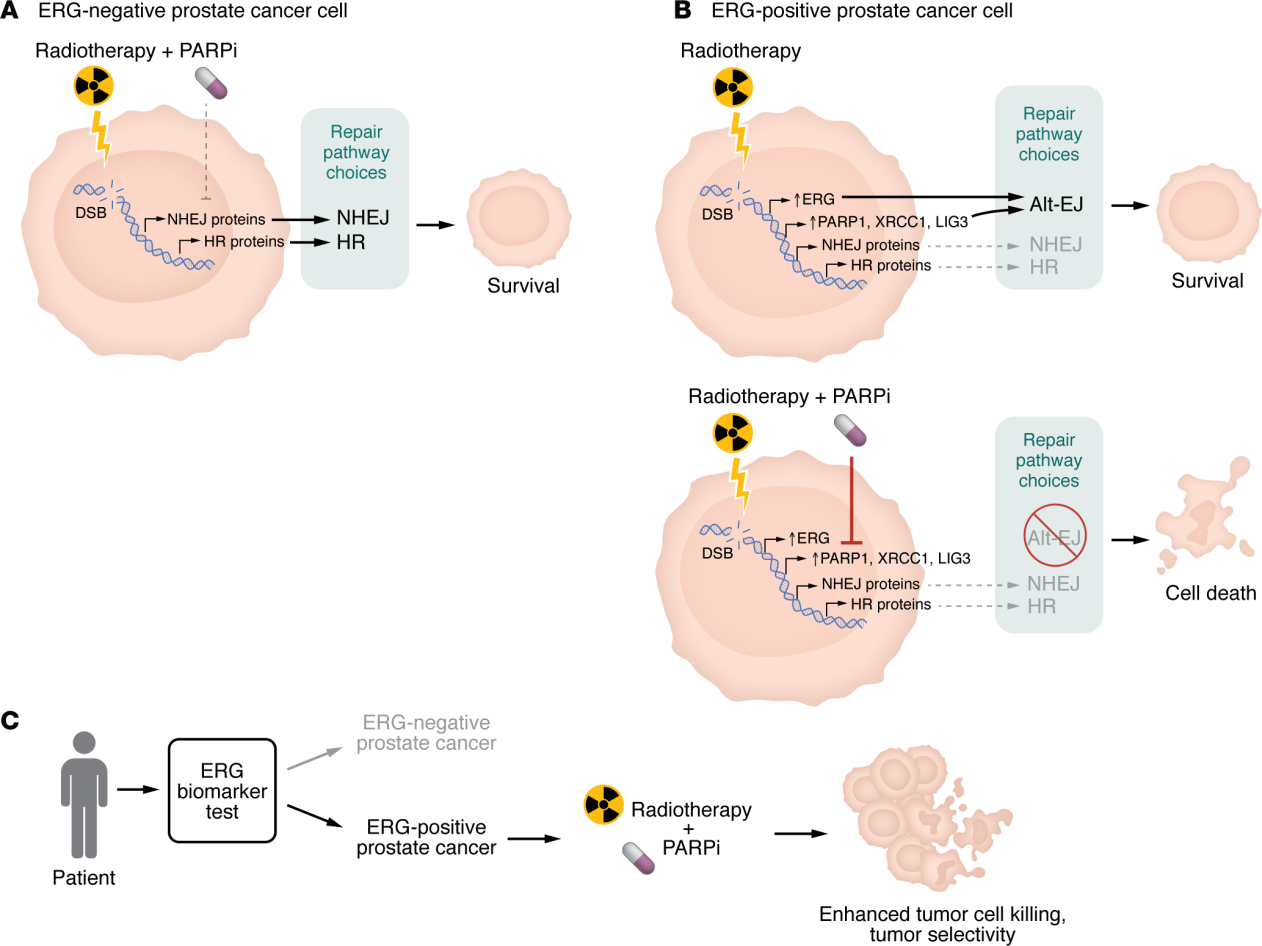
ERG-driven DNA repair rewiring creates a therapeutic window for radiosensitization. Köcher et al. (8) showed that the *TMPRSS2:ERG* fusion reprograms DNA double-strand break (DSB) repair in prostate cancer to create a selective vulnerability to PARP inhibitor–mediated radiosensitization. (**A**) In ERG-negative cells, radiation-induced DSBs are predominantly repaired by canonical nonhomologous end-joining (NHEJ) and homologous recombination (HR), and PARP inhibition (PARPi) has minimal impact. (**B**) In contrast, ERG overexpression shifts DSB repair toward PARP1-dependent alternative end-joining (Alt-EJ) without impairing HR or NHEJ. Upon PARPi during radiotherapy, ERG-positive cells lose their dominant repair pathway, resulting in persistent γH2AX/53BP1 foci, genomic instability, and enhanced tumor cell death. (**C**) Thus, ERG expression serves as a predictive biomarker for tumor-selective radiosensitization.

Among genomic alterations identified in prostate cancer, rearrangements involving ETS family transcription factors are the most prevalent. Fusion of the androgen-responsive *TMPRSS2* promoter to the *ERG* oncogene occurs in approximately 50% of prostate cancers in patients of European ancestry (5–7). Although the prognostic significance of *TMPRSS2:ERG* has remained controversial, growing evidence indicates that ERG overexpression imposes distinct biological states and therapeutic dependencies. The study by Köcher et al. (8) provides compelling mechanistic and translational evidence that ERG overexpression reprograms DNA double-strand break (DSB) repair pathway choice, creating a selective vulnerability to poly(ADP-ribose) polymerase inhibition (PARPi) in the context of RT. Collectively, these findings reposition *TMPRSS2:ERG* from a debated prognostic marker to a functional biomarker for tumor-selective radiosensitization (Figure 1).

## ERG as a truncal oncogenic driver

*ERG* rearrangements represent truncal events in prostate tumorigenesis, arising early and persisting throughout disease evolution (6). By placing *ERG* under the control of the androgen-responsive *TMPRSS2* promoter-enhancer, this rearrangement enforces sustained *ERG* expression throughout disease evolution. Unlike androgen receptor (AR) alterations — which typically emerge under selective pressure from hormonal therapies — *ERG* gene rearrangements precede treatment and define a molecular subtype from tumor initiation onward (9).

ERG expression is absent from normal prostate epithelium, underscoring its tumor specificity (10). This distinction has important therapeutic implications: ERG-driven dependencies are unlikely to be shared by surrounding normal tissues, creating opportunities for selective intervention. Despite its high prevalence, ERG has proven difficult to directly target. As a transcription factor, ERG lacks enzymatic activity, and early efforts to use ERG as a prognostic biomarker yielded inconsistent results across prostatectomy and RT cohorts (11).

These inconsistencies suggest that ERG does not function as a classical prognostic determinant. Instead, ERG defines a molecular subtype with distinct responses to cellular stress, including DNA damage induced by therapy. Functionally, ERG orchestrates transcriptional programs linked to invasion (7), lineage plasticity, and therapeutic resistance (12), and emerging evidence places ERG at the intersection of genome stability and DNA repair pathway regulation (13).

## The ERG axis rewires DNA damage responses

RT exerts its antitumor effects primarily by inducing DNA DSBs, and the efficiency and fidelity of DSB repair are major determinants of radiosensitivity (14). In mammalian cells, DSBs are repaired predominantly through canonical nonhomologous end-joining (c-NHEJ) and homologous recombination (HR). Defects in HR, such as those caused by BRCA1/2 mutations, confer sensitivity to PARP inhibitors and form the basis of their current clinical use (15). However, such defects are relatively uncommon in prostate cancer, limiting the applicability of established PARPi paradigms (16).

In addition to HR and c-NHEJ, DSBs can be repaired by an alternative end-joining (Alt-EJ) pathway. Alt-EJ is typically engaged when c-NHEJ is compromised and is characterized by its reliance on PARP1 (PARP1-dependent end-joining; PARP1-EJ) and its intrinsically error-prone nature (17). Cells that shift DSB repair toward PARP1-EJ become selectively vulnerable to PARPi, particularly in the setting of radiation-induced DNA damage (18). Importantly, this mode of radiosensitization is replication independent and does not require preexisting HR deficiency, distinguishing it from canonical PARPi sensitivity.

In the present study, Köcher et al. (8) demonstrated that ERG overexpression in human prostate cancer cell lines did not abolish canonical DSB repair. ERG-positive prostate cancer cells retained intact HR and c-NHEJ activity, consistent with clinical data showing comparable outcomes following RT alone in ERG-positive and ERG-negative tumors (11). Rather than inducing repair defects, ERG drove a qualitative shift in repair pathway choice: ERG-positive cells preferentially engaged PARP1-EJ, supported by increased expression and utilization of PARP1, XRCC1, and DNA ligase III, instead of DNA-PK–dependent c-NHEJ (19). Sequencing of repair junctions revealed increased deletions and imprecise end-joining, molecular hallmarks of PARP1-EJ–mediated repair.

Thus, ERG rewires DSB repair architecture without reducing overall repair capacity, creating a conditional dependency on PARP1 that becomes apparent only under genotoxic stress.

## Mechanistic basis of PARP dependency in ERG-positive tumors

Mechanistically, ERG functions as a transcriptional organizer of the PARP1-EJ axis. Köcher et al.’s proteomic and transcriptional analyses revealed upregulation of PARP1-EJ components, including PARP1, XRCC1, LIG3, and MRE11, in ERG-positive cells (8). ERG depletion reversed this expression pattern, consistent with ERG’s role as a chromatin-binding transcriptional regulator (20).

This ERG-driven program biases DSB repair toward an error-prone but flexible pathway capable of accommodating the high transcriptional output and replication stress characteristic of ERG-positive tumors (21). In this context, PARP1-EJ may represent an adaptive solution for genome maintenance — until therapeutic pressure is applied.

PARPi alone had minimal effects on cell survival or DNA repair in ERG-positive cells. However, when combined with radiation-induced DSBs, PARPi removed the dominant repair pathway on which these cells rely, resulting in persistent unrepaired damage and marked radiosensitization.

## ERG as a predictive biomarker for precision RT

The therapeutic significance of ERG-mediated repair rewiring becomes evident when PARPi is combined with RT. In the absence of irradiation, PARP inhibitors exerted limited cytotoxicity in ERG-positive prostate cancer cells, consistent with intact HR and c-NHEJ (22). Upon radiation exposure, however, inhibition of PARP1 abolished the preferred repair pathway, leading to accumulation of unrepaired DSBs marked by persistent γH2AX and 53BP1 foci and subsequent tumor cell death (23).

This radiosensitization was selective: ERG-negative cells, which rely primarily on canonical repair pathways, were largely spared. Patient-derived organoid models corroborated these findings, demonstrating that combined PARPi and irradiation selectively suppressed growth and survival of ERG-positive organoids, while ERG-negative organoids derived minimal benefit.

## Clinical implications and future directions

Identifying the *TMPRSS2:ERG* fusion as a biomarker for PARPi-mediated radiosensitization represents a conceptual advance in precision RT. Unlike BRCA mutations, which occur in a minority of patients, ERG fusion is present in nearly half of prostate cancers, substantially expanding the population that could benefit from targeted radiosensitization.

Clinically, combining PARP inhibitors with RT in ERG-positive patients offers several advantages: tumor selectivity due to absent ERG expression in normal tissues, transient PARPi exposure limited to the RT window to minimize toxicity and resistance (24), and straightforward patient stratification using existing diagnostics. Effective radiosensitization may also permit treatment de-escalation, potentially reducing the duration or intensity of ADT and improving quality of life without compromising tumor control (25).

Prospective clinical trials are now warranted to validate ERG-guided combination strategies and to optimize dosing, scheduling, and patient selection. Incorporation of correlative studies will be essential to further elucidate the molecular circuitry linking ERG, PARP1-EJ, and DNA damage signaling.

## Conclusion

The *TMPRSS2:ERG* fusion defines a biologically distinct and therapeutically actionable subtype of prostate cancer. By rewiring DSB repair toward PARP1-EJ, ERG overexpression creates a selective vulnerability that can be exploited using PARP inhibitors in combination with RT. These findings shift the paradigm of ERG from a debated prognostic marker to a functional biomarker for precision RT, offering a rational strategy to enhance tumor control while minimizing normal tissue toxicity.

## Funding support

This work is the result of NIH funding, in whole or in part, and is subject to the NIH Public Access Policy. Through acceptance of this federal funding, the NIH has been given a right to make the work publicly available in PubMed Central.

- NIH National Cancer Institute (NCI) Prostate Specialized Program of Research Excellence (SPORE) Grant (2P50CA186786-11A1, AMC).
- NCI Early Detection Research Network (U2C-CA271854, AMC).
- NCI Outstanding Investigator Award (R35-CA231996, AMC).
- Howard Hughes Medical Institute Investigator, Alfred Taubman Scholar, and American Cancer Society Professor (AMC).

## Version 1 03/16/2026

Electronic publication
